# Caveolin-1 and Sox-2 are predictive biomarkers of cetuximab response in head and neck cancer

**DOI:** 10.1172/jci.insight.151982

**Published:** 2021-10-22

**Authors:** Mehdi Bouhaddou, Rex H. Lee, Hua Li, Neil E. Bhola, Rachel A. O’Keefe, Mohammad Naser, Tian Ran Zhu, Kelechi Nwachuku, Umamaheswar Duvvuri, Adam B. Olshen, Ritu Roy, Aaron Hechmer, Jennifer Bolen, Stephen B. Keysar, Antonio Jimeno, Gordon B. Mills, Scott Vandenberg, Danielle L. Swaney, Daniel E. Johnson, Nevan J. Krogan, Jennifer R. Grandis

**Affiliations:** 1 Department of Cellular and Molecular Pharmacology and; 2 Quantitative Biosciences Institute, University of California, San Francisco, San Francisco, California, USA.; 3 J. David Gladstone Institutes, San Francisco, California, USA.; 4 Department of Otolaryngology — Head and Neck Surgery and; 5 Histology and Biomarkers Core, Helen Diller Family Comprehensive Cancer Center Biorepository and Tissue Biomarker Technology, University of California, San Francisco, San Francisco, California, USA.; 6 Department of Otolaryngology and UPMC Hillman Cancer Center, University of Pittsburgh, Pittsburgh, Pennsylvania, USA.; 7 Computational Biology and Informatics Core and; 8 Department of Epidemiology and Biostatistics, University of California, San Francisco, San Francisco, California, USA.; 9 Department of Medicine, University of Colorado Hospital, Aurora, Colorado, USA.; 10 Knight Cancer Institute, Oregon Health and Sciences University, Portland, Oregon, USA.

**Keywords:** Therapeutics, Cancer

## Abstract

The epidermal growth factor receptor (EGFR) inhibitor cetuximab is the only FDA-approved oncogene-targeting therapy for head and neck squamous cell carcinoma (HNSCC). Despite variable treatment response, no biomarkers exist to stratify patients for cetuximab therapy in HNSCC. Here, we applied unbiased hierarchical clustering to reverse-phase protein array molecular profiles from patient-derived xenograft (PDX) tumors and revealed 2 PDX clusters defined by protein networks associated with EGFR inhibitor resistance. In vivo validation revealed unbiased clustering to classify PDX tumors according to cetuximab response with 88% accuracy. Next, a support vector machine classifier algorithm identified a minimalist biomarker signature consisting of 8 proteins — caveolin-1, Sox-2, AXL, STING, Brd4, claudin-7, connexin-43, and fibronectin — with expression that strongly predicted cetuximab response in PDXs using either protein or mRNA. A combination of caveolin-1 and Sox-2 protein levels was sufficient to maintain high predictive accuracy, which we validated in tumor samples from patients with HNSCC with known clinical response to cetuximab. These results support further investigation into the combined use of caveolin-1 and Sox-2 as predictive biomarkers for cetuximab response in the clinic.

## Introduction

The incidence of head and neck squamous cell carcinoma (HNSCC) is increasing, with few FDA-approved targeted therapy options. Cetuximab, a monoclonal antibody directed against the epidermal growth factor receptor (EGFR), was approved by the FDA in 2006 as the first molecular targeted agent for HNSCC. It performed favorably in a series of clinical trials, leading to approval in combination with radiation as first-line therapy for locoregionally advanced HNSCC, in conjunction with platinum-based therapy and fluorouracil for recurrent or metastatic disease, or as a single agent in recurrent or metastatic disease refractory to platinum-based therapy (1–3). However, the variability in clinical response to cetuximab limits its clinical use. To date, no biomarker, including expression, copy number, or phosphorylation of EGFR itself, has consistently correlated with clinical response to cetuximab in HNSCC (4–6).

A major obstacle in defining molecular characteristics predictive of cetuximab response is the limitation of HNSCC cell line models for informing clinical decisions. The genomic profiles of many immortalized cell lines are highly divergent from those of excised patient samples, with cell lines harboring mutations arising from time in culture that are absent from primary tumors (7). In contrast, xenograft tumors derived directly from patient samples are more representative of in vivo human biology. Proteomic analysis of patient-derived xenografts (PDXs) has shown that HNSCC PDXs are far more similar to human primary tumors than are HNSCC cell lines or xenografts derived from cell lines (8). Thus, the use of HNSCC PDXs provides an opportunity to more accurately recapitulate human tumor biology in the preclinical setting, with greater promise to guide clinical decision making. Characterizing the molecular tumor profiles that correlate with cetuximab response in PDX models of HNSCC may enable the identification of biomarkers for treatment stratification.

Here, we report predictive biomarkers of cetuximab response derived from reverse-phase protein array (RPPA) assessment of 247 markers across a cohort of 65 HNSCC PDXs. An unsupervised clustering analysis separated HNSCC PDXs into 2 distinct clusters defined by differential expression of proteins implicated in EGFR inhibitor resistance. We next developed a support vector machine (SVM) classifier algorithm in order to define a minimalist biomarker signature able to predict in vivo treatment response. We identified an 8-gene signature able to predict cetuximab response using either protein or mRNA measurements, which we validated on an independent HNSCC PDX cohort (9). Finally, histopathological analysis of samples from patients with HNSCC revealed that high caveolin-1 and low Sox-2 was predictive of clinical response to cetuximab, whereas low caveolin-1 and high Sox-2 was predictive of intrinsic cetuximab resistance. These findings motivate the combined use of caveolin-1 and Sox-2 as biomarkers to stratify patients with HNSCC for cetuximab therapy.

## Results

### Unbiased clustering of protein biomarkers reveals 2 PDX subtypes.

**Figure 1:**
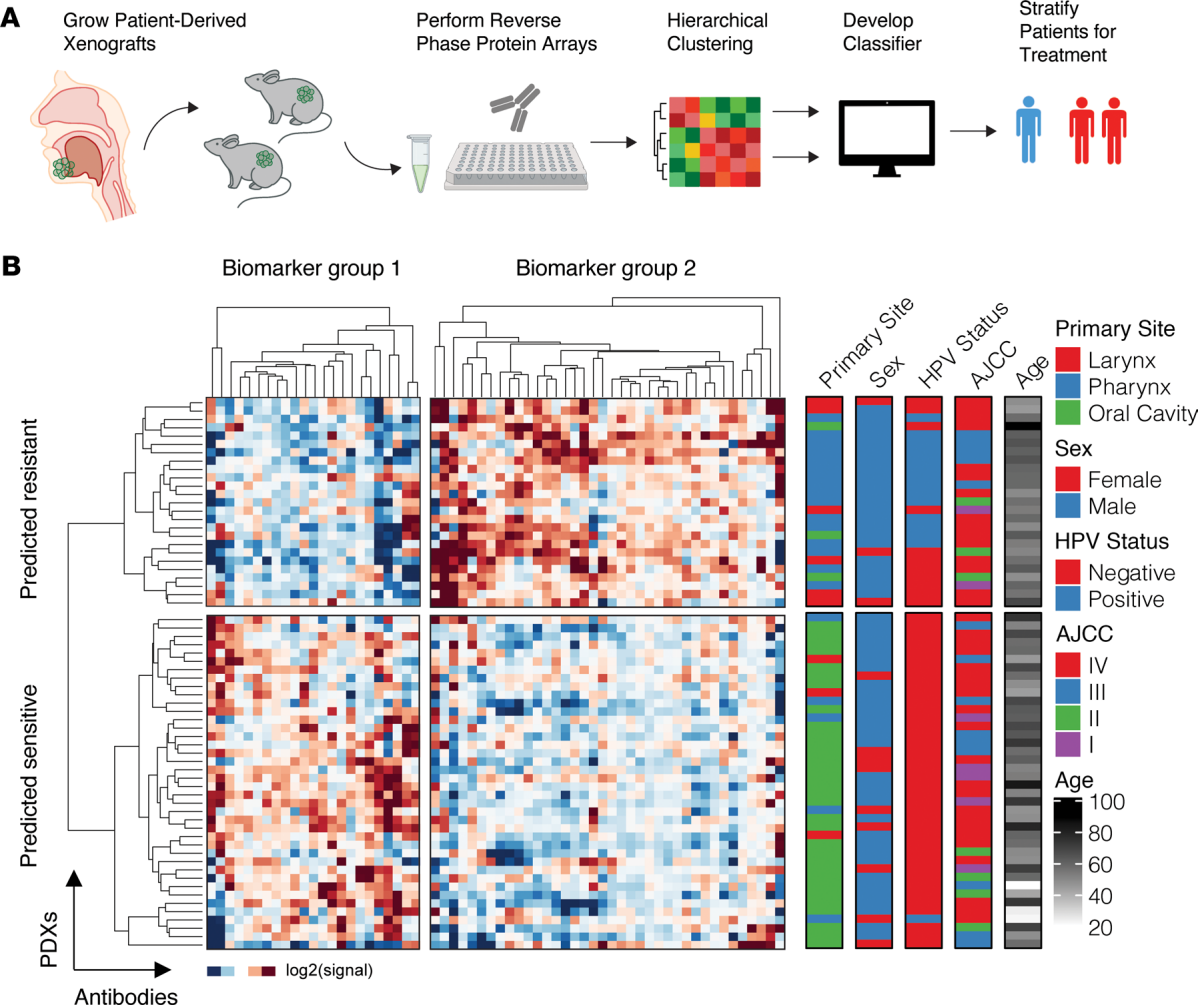
Hierarchical clustering of protein markers reveals 2 HNSCC PDX subtypes. (**A**) Schematic of PDX derivation, reverse-phase protein array (RPPA), and clustering analysis. Primary patient HNSCC tumors were excised and implanted into NOD/SCID γ mice. Lysates of the xenografted cells were molecularly profiled using RPPA for 247 proteins and then analyzed via an unsupervised hierarchical clustering analysis. (**B**) Unbiased hierarchical clustering yields 2 PDX clusters (rows) defined by differential expression of candidate biomarker groups 1 and 2 (columns). Primary site, sex, HPV status, American Joint Committee on Cancer (AJCC) tumor stage, and age of patients in years are indicated (right). Signal is defined as the median-centered chemiluminescent signal from the RPPA assay.

**Figure 2:**
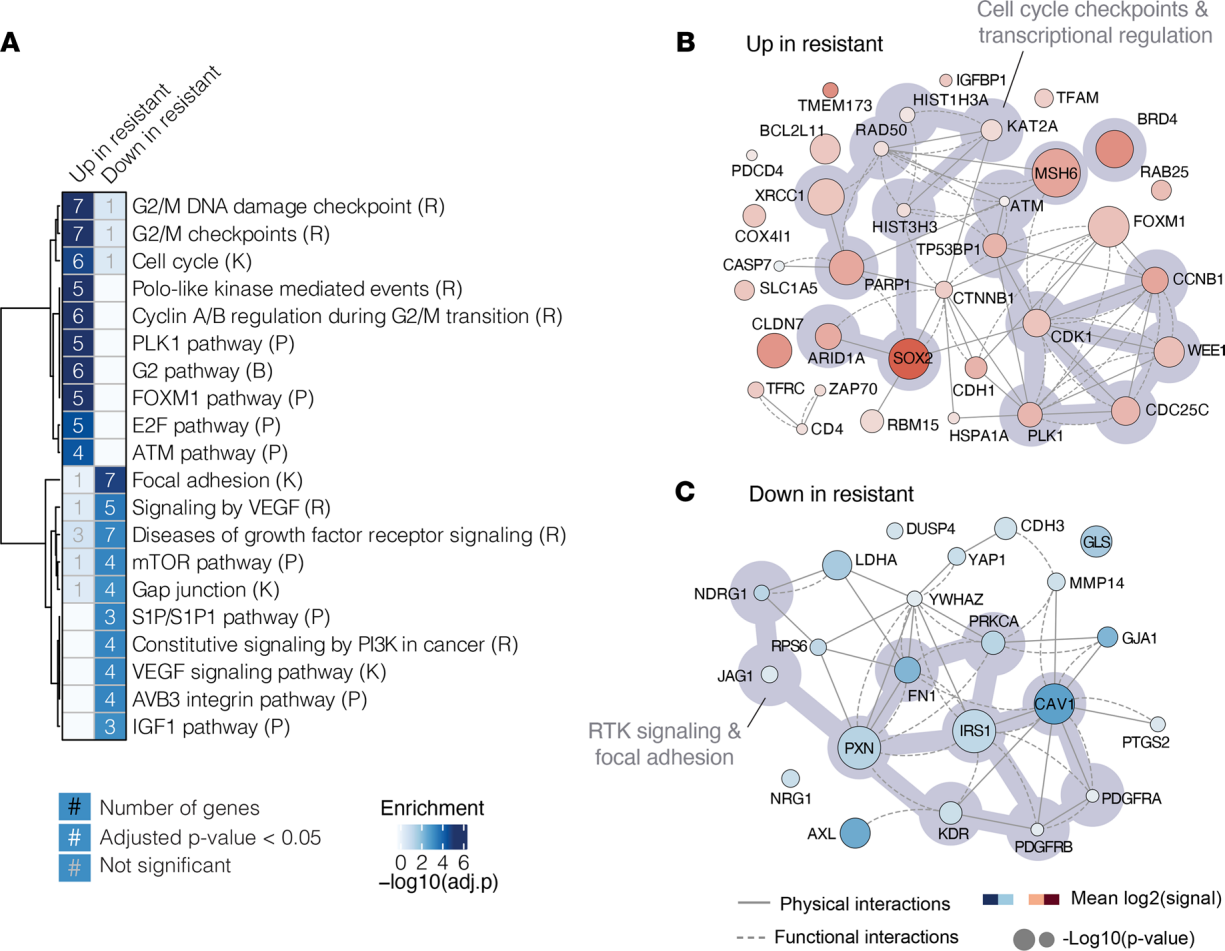
Biological description of biomarker groups. (**A**) MsigDB gene set enrichment analysis using Reactome (R), KEGG (K), Biocarta (B), and PID (P) pathways (extracted from the MSigDB repository) for genes underlying biomarker group 1 and 2. (**B**) Physical and functional interaction network of genes upregulated in the predicted resistant PDX cluster (biomarker group 2). Functional interactions from ReactomeFI and physical interactions from Bioplex, IRefIndex, Mentha, HumanInteractome, and Human Protein Reference Database (HPRD). (**C**) As in **B**, physical and functional interaction network of genes downregulated in the predicted resistant PDX cluster (biomarker group 1). The size of the circles is proportional to the –log_10_(*P* value) derived from a Student’s *t* test comparing 2 patient groups revealed by hierarchical clustering in Figure 1.

RPPA analysis of 247 total and phospho-protein levels was performed on 65 HNSCC PDXs grown subcutaneously in NOD/SCID γ mice (Supplemental Tables 1 and 2; supplemental material available online with this article; https://doi.org/10.1172/jci.insight.151982DS1), as described previously (8). An unsupervised hierarchical clustering approach was used to group the PDXs based on their protein expression signatures (Figure 1A, Supplemental Figure 1A, and Supplemental Table 3), which yielded 2 distinct PDX clusters defined by 2 biomarker clusters (Figure 1B; Supplemental Figure 1, B and C; and Supplemental Table 3). Gene set enrichment analysis of genes within each biomarker cluster revealed that 1 cluster (biomarker group 1) was enriched for cell cycle processes, DNA repair, and transcription regulation (Figure 2, A and B), while the other cluster (biomarker group 2) was enriched for was enriched for RTK signaling, MAPK and PI3K pathways, and extracellular matrix organization, highlighting regulation of gap junctions and focal adhesions (Figure 2, A and C).

The higher signal intensity of pathways downstream from RTKs in the lower PDX cluster, such as the MAPK and PI3K pathways (biomarker group 1), suggested a possible dependence on RTK-mediated signaling, perhaps via mechanisms of oncogene addiction (10), and potentially increased sensitivity to EGFR inhibition. Conversely, higher signal intensity of cell cycle and transcriptional processes (biomarker group 2) in the upper PDX cluster suggested control of cellular proliferation that may be independent of upstream RTK signaling. Furthermore, heightened expression of Sox-2 and BRD4 in the upper PDX cluster further supported a phenotype of EGFR inhibitor resistance (11–15). These observations led us to hypothesize that PDXs in the lower and upper clusters would be cetuximab sensitive and resistant, respectively (Figure 1B).

### PDX cluster membership aligns with cetuximab response.

**Figure 3:**
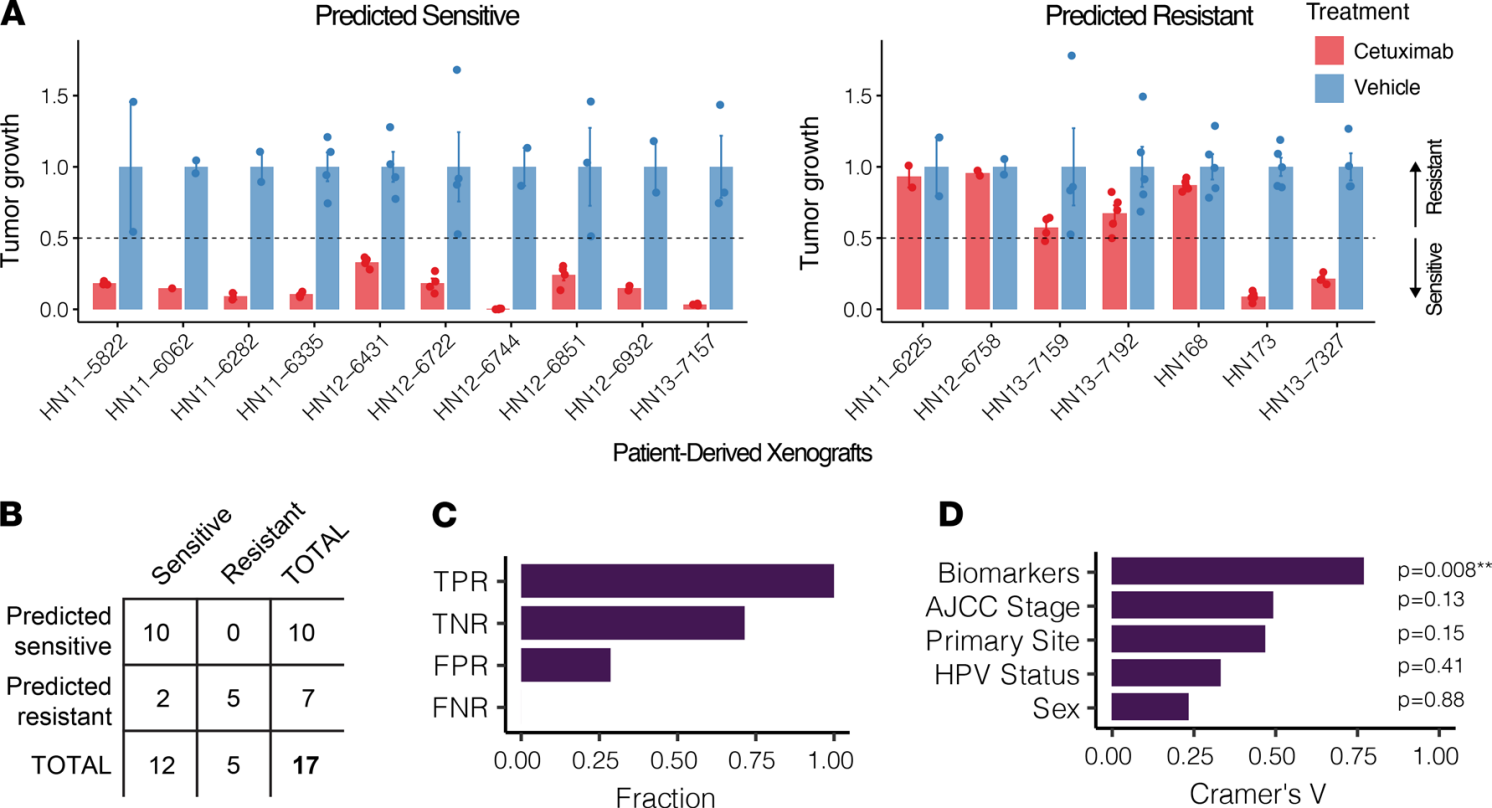
In vivo cetuximab treatment efficacy aligns with predicted PDX cetuximab response. (**A**) Experimental endpoint average tumor volume after cetuximab treatment (normalized to vehicle) for 17 PDXs: 10 predicted sensitive (left) and 7 predicted resistant (right). We defined sensitivity as 50% or greater reduction in endpoint tumor volume relative to vehicle (black dashed line). Error bars depict the SEM. There were between 2 and 3 animals used per PDX (dots). (**B**) Contingency table detailing the accuracy of hierarchical clustering-based protein biomarkers (from Figure 1B) in predicting cetuximab response. 10 of 10 of implanted PDXs predicted to be sensitive responded to cetuximab (100% accuracy), whereas 5 of 7 of the PDXs that were predicted to be resistant demonstrated cetuximab resistance (71% accuracy). (**C**) True positive rate (TPR), true negative rate (TNR), false negative rate (FNR), and false positive rate (FPR) percentages from **B**. Positive was defined as sensitive to cetuximab. (**D**) Cramér’s V analysis assessing the strength of association between cetuximab response and biomarkers, HPV status, American Join Committee on Cancer (AJCC) stage, sex, or primary tumor site of the experimentally validated PDXs in **A**. The significance of each association (i.e., *P* value) was determined using a Pearson’s χ^2^ test. **0.001 < *P* < 0.01.

We next sought to test our hypothesis by comparing our predictions to the in vivo cetuximab response of PDXs from each cluster. Seven PDXs predicted to be cetuximab resistant and ten PDXs predicted to be cetuximab sensitive (Figure 3A and Supplemental Table 4) were implanted into NOD/SCID γ mice, and once the tumor volume reached approximately 200 mm^3^, the mice were administered intraperitoneal injections of cetuximab or vehicle twice weekly for 20 days. Tumors demonstrating a 50% or greater reduction in tumor volume (relative to vehicle) on the final day of treatment were considered cetuximab sensitive; the rest were considered cetuximab resistant. All 10 of the PDXs predicted to be sensitive responded strongly to cetuximab, resulting in 100% accuracy (95% confidence interval of 69%–100%). Five of the seven PDXs predicted to be resistant displayed resistance, resulting in 71% accuracy (95% confidence interval of 30%–96%) (Figure 3, A and B). In total, hierarchical clustering of protein markers successfully predicted in vivo cetuximab response for 15 of 17 samples, resulting in 88% accuracy (95% confidence interval of 64%–99%), a true positive rate of 100%, a true negative rate of 71%, a false positive rate of 29%, and a false negative rate of 0% (Figure 3, B and C). Although HPV status correlated well with cetuximab response, consistent with recent landmark trials showing poor response of HPV-positive HNSCC to cetuximab (16, 17), a Cramér’s V analysis revealed that protein biomarker-based clustering outperformed HPV status, American Joint Committee on Cancer clinical stage, sex, and primary site in predicting in vivo cetuximab response in the PDX samples, and it was the only significant correlate based on a χ^2^ test (Figure 3D).

### Defining a robust biomarker signature for cetuximab response.

**Figure 4:**
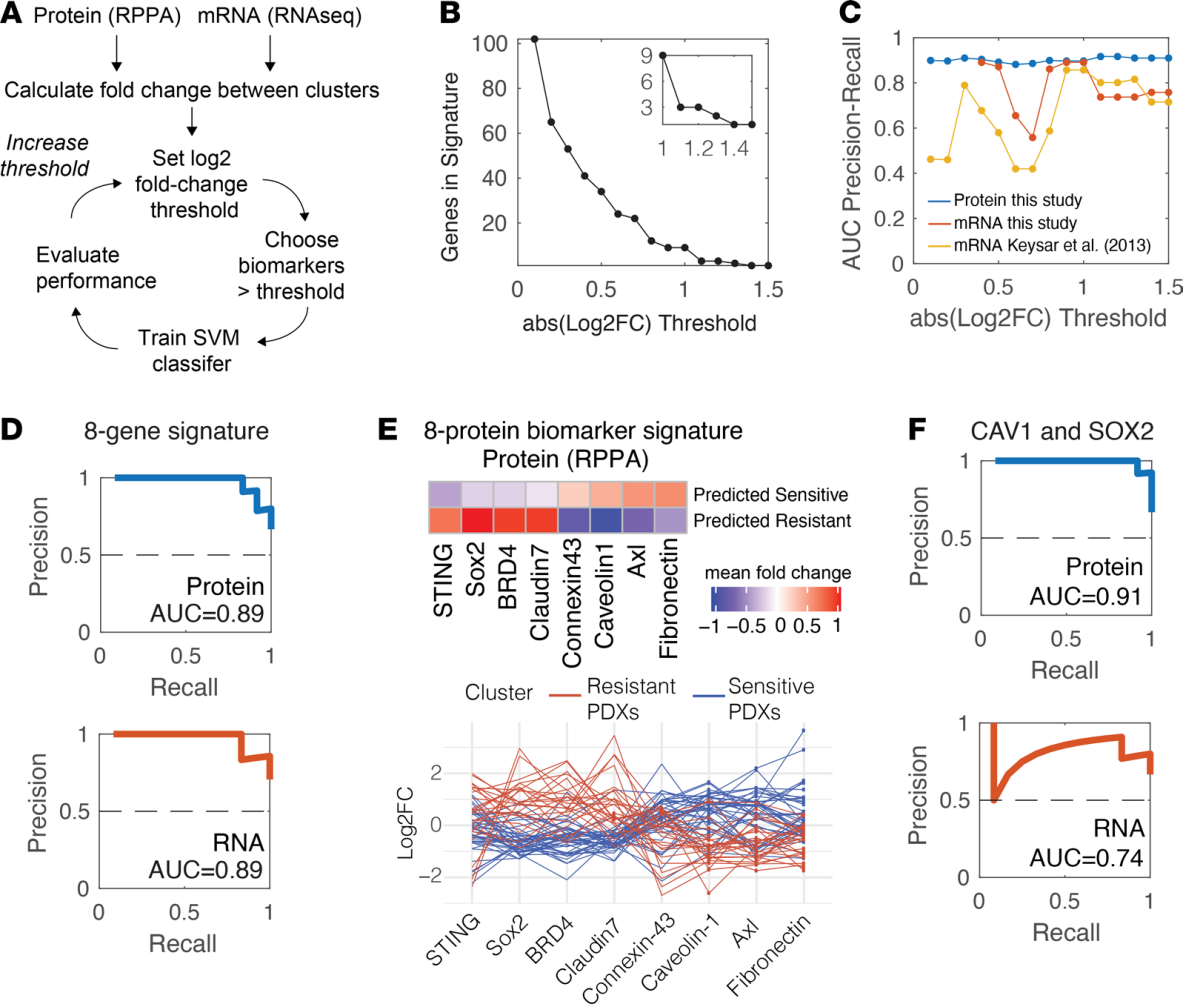
Defining a robust signature for cetuximab response. (**A**) Schematic of algorithmic approach for identifying and testing biomarker sets for cetuximab response. (**B**) The number of biomarker reverse-phase protein array (RPPA) probes at each successive absolute value log_2_ fold change (FC) threshold in 0.1 increments, which defined 15 distinct biomarker sets. The inset shows FC thresholds greater than 1. (**C**) Area under the precision-recall curves evaluating support vector machine (SVM) classifier performance of each of 15 biomarker sets defined in **B** for protein (RPPA; blue) or mRNA (RNA-Seq; red). SVM classifiers were trained based on hierarchical clustering results (Figure 1B) for PDXs not experimentally validated for cetuximab response and then tested on those that were experimentally evaluated. An independent cohort of PDXs was also evaluated (RNA-Seq; yellow; Keysar et al, ref. 9). (**D**) Precision-recall curves for the 8-member biomarker set defined at a log_2_FC threshold of 1, where predictive accuracy is high for both protein- and mRNA-based measurements. (**E**) (Top) Heatmap of protein (RPPA) mean signal intensities for 8-member biomarker set defined at a log_2_FC threshold of 1. (Bottom) Parallel coordinates chart displaying protein (RPPA) intensities of 8-member biomarker set displaying median-normalized protein log_2_FC for the predicted resistant (red) or sensitive (blue) groups. (**F**) Precision-recall curves for the top 2 biomarkers from **E**, caveolin-1 and Sox-2.

Next, we sought to define a minimalist biomarker signature for cetuximab response that could be used to stratify patients with HNSCC for cetuximab therapy. We devised an algorithm that prioritized biomarkers with the largest differences between the 2 PDX subgroups. We first calculated the signal intensity fold change between the cetuximab-resistant and -sensitive clusters for each protein biomarker (Figure 4A). By imposing incremental fold change thresholds (absolute value of log_2_FC), we defined 15 distinct protein (RPPA) biomarker sets, each with successively fewer proteins (Figure 4B). To evaluate the predictive power of each candidate biomarker set, we trained an SVM classifier against the predicted sensitive and resistant clusters, including all PDXs except the 17 that were experimentally validated. We then tested the classifying performance against cetuximab response for the 17 validated tumors. Briefly, an SVM creates an n-dimensional hyperplane that differentiates between 2 defined groups. Classifier performance for each fold change threshold was evaluated by measuring the areas under the precision-recall curves (Figure 4C, blue).

**Figure 5:**
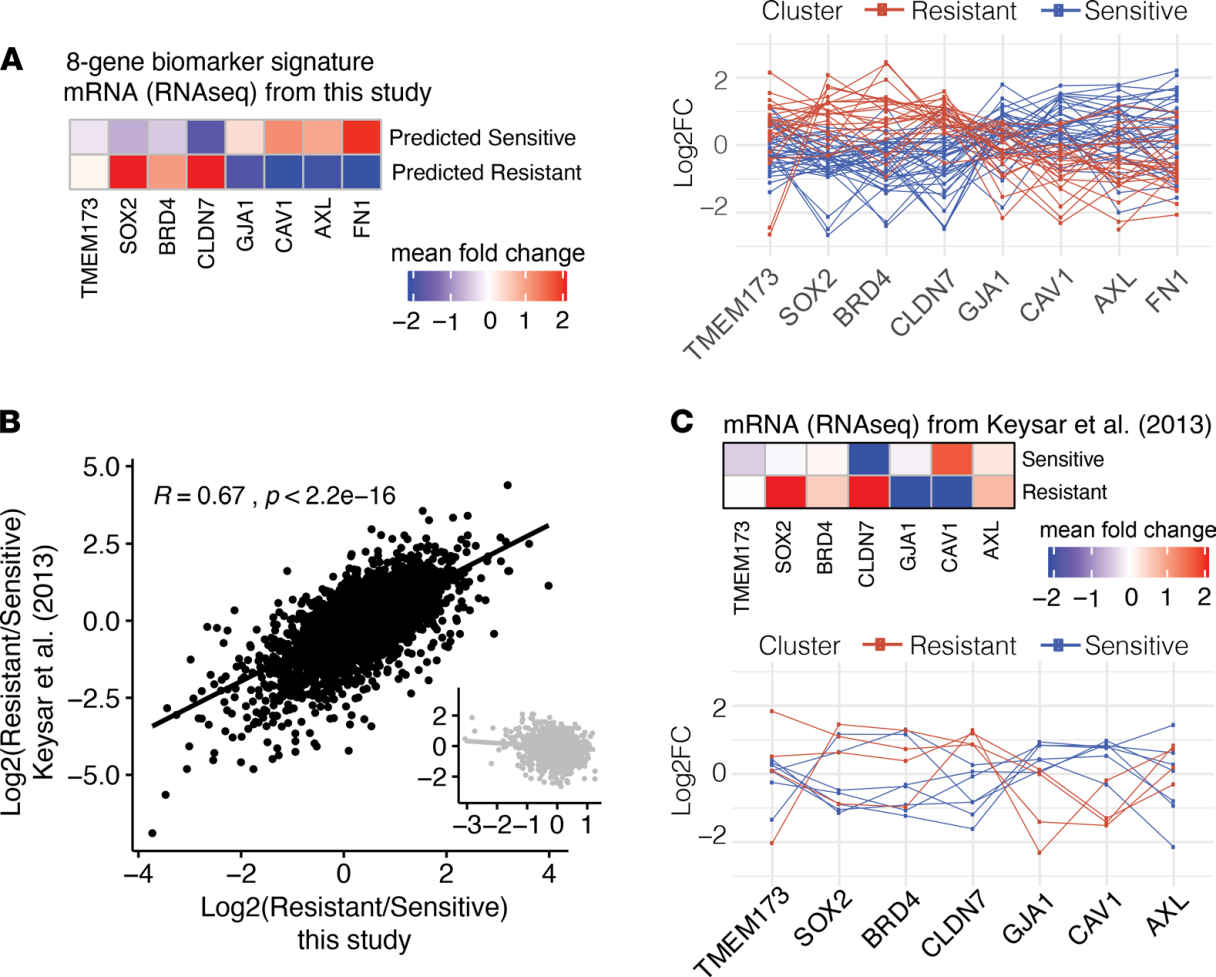
Validation of 8-member biomarker signature in mRNA data sets. (**A**) Heatmap of mRNA mean signal intensities for 8-member biomarker set defined at log_2_FC threshold of 1, and parallel coordinates chart displaying median-normalized mRNA log_2_FC of the 8-member biomarker set for each individual PDX of the predicted resistant (red) or sensitive (blue) groups. (**B**) Fold changes (log_2_FC) of mRNA levels between the predicted resistant and predicted sensitive groups in this study versus analogous fold changes from Keysar et al. (9) (*R* = 0.67, *P* < 2.2 × 10^–16^). The inset shows patients randomized to either cetuximab-sensitive or -resistant groups prior to calculating the fold change in order to control for the possibility that any grouping would result in a correlation between the studies. (**C**) (Top) Heatmap of mRNA mean signal intensities for 7 genes of the original 8-member biomarker set, defined at log_2_FC threshold of 1 from Keysar et al. (9) (FN1 was not present in the Keysar et al. study). (Bottom) Parallel coordinates chart displaying median-normalized mRNA log_2_FC of 7-member biomarker set for each individual PDX of the resistant (red) or sensitive (blue) groups.

To assess the generalizability of this protein signature to mRNA measurements, the same 65 PDX samples were subjected to global mRNA sequencing (see Methods and Supplemental Table 5). The same 15 biomarker sets were then evaluated for their predictive power using normalized mRNA measurements as the test set; the training set remained the RPPA protein-based measurements (Figure 4C, red). We found that an absolute value log_2_FC threshold of 1 possessed the best SVM classifier performance for both protein -and mRNA-based measurements (Figure 4C), revealing a 9-member biomarker set (Figure 4B, inset). To restrict our biomarker set to proteins measurable by both mRNA and protein, we removed the PAR probe (which detects poly(ADP-ribose) polymer), resulting in an 8-member biomarker signature for cetuximab response: 4 overexpressed in cetuximab-sensitive tumors (connexin-43 [*GJA1*], caveolin-1 [*CAV1*], AXL, and fibronectin [*FN1*]) and 4 overexpressed in cetuximab-resistant tumors (STING [*TMEM173*], Sox-2 [*SOX2*], BRD4, and claudin-7 [*CLDN7*]) (Figure 4, D and E, and Figure 5A). Importantly, although the expression of individual candidates was clearly correlated with cluster membership, no single protein/gene was able to perfectly distinguish clusters, as variability was observed between PDXs (Figure 4E, bottom). The top 2 biomarkers, caveolin-1 and Sox-2, retained high predictive power for protein-based (AUC = 0.91), but not mRNA-based (AUC = 0.74), measurements (Figure 4F), suggesting that these could be good predictive biomarkers for clinical use where immunohistochemistry-based approaches are more easily implemented than those requiring the extraction of mRNA.

### Validation of biomarker signature on an independent PDX cohort.

To safeguard against the possibility of model overfitting, we sought to evaluate our model’s performance on an independent PDX data set by comparing the concordance of mRNA measurements from our biomarker signature with those of other experimentally validated PDXs treated with cetuximab (Figure 5A). Specifically, we extracted transcriptomic measurements from 4 cetuximab-resistant and 7 cetuximab-sensitive PDXs from the 2013 study by Keysar et al. (9). Fold changes (log_2_FC) in mRNA levels between the predicted resistant and predicted sensitive groups in this study correlated well with analogous fold changes from Keysar et al. (9) (*R* = 0.67, *P* < 2.2 ×10^–16^; Figure 5B). To control for the possibility that any grouping would result in a correlation between the studies, we randomized PDXs labeled as sensitive or resistant and found that the correlation was abrogated (Figure 5B, inset).

Next, as performed above for the mRNA-sequencing data from our PDX cohort, we passed the Keysar et al. (9) mRNA expression levels as inputs into the SVM models trained against our cluster-defined RPPA data. At each fold change threshold, model performance was evaluated and area under the precision-recall curve was calculated (Figure 4C, yellow). The biomarker set identified at a fold change threshold of 1 resulted in the highest predictive accuracy for cetuximab response in the Keysar et al. (9) data set, in agreement with the mRNA-based results from our PDX cohort (Figure 4C, red). (Of the 8 markers present in our biomarker signature, only 7 were also measured by Keysar et al. (9): *TMEM173*, *SOX2*, *BRD4*, *CLDN7*, *GJA1*, *CAV1*, and *AXL.*) Differential mean expression between confirmed resistant and sensitive PDXs corresponded to the patterns seen in our RPPA and RNA-Seq data set for all transcripts, with the exception of *AXL*, which was upregulated in resistant PDXs from Keysar et al. (9) (Figure 5C).

### Validation of biomarker signature in cetuximab-treated HNSCC clinical cohort.

**Figure 6:**
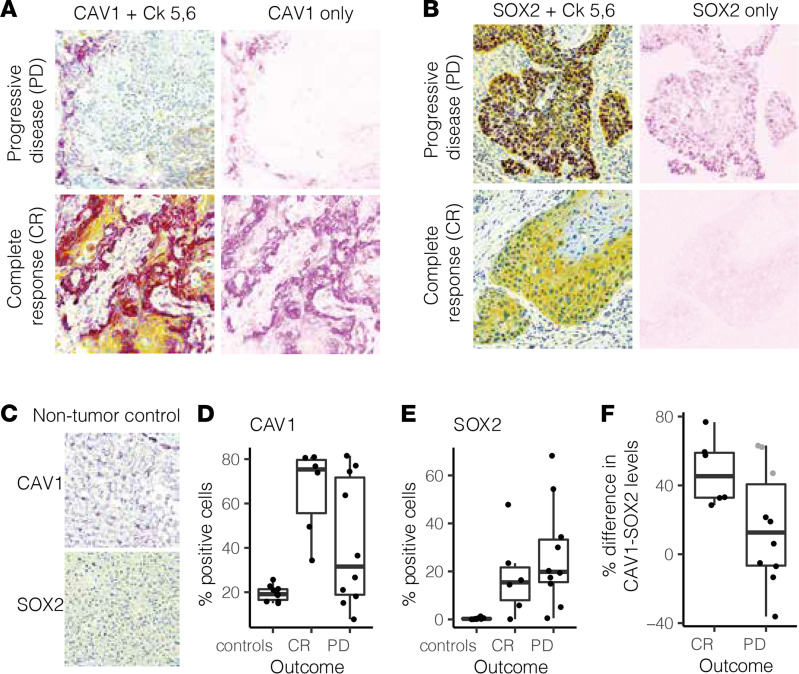
Validation of caveolin-1 and Sox-2 as biomarkers in HNSCC clinical samples from patients with known clinical responses to cetuximab. (**A**) Representative images of tumor sections from patients with progressive disease (PD; top) or complete response (CR; bottom), proxies for resistance and sensitivity, respectively. Cells are stained for caveolin-1 (purple), cytokeratin 5,6 (yellow), and DNA (blue). Image deconvolution was applied computationally to extract the caveolin-1–only part of the image. (**B**) Representative images of tumor sections from patients with progressive disease (top) or complete response (bottom), proxies for resistance and sensitivity, respectively. Cells were stained for Sox-2 (purple), keratin (yellow), and DNA (blue). Image deconvolution was applied computationally to extract the Sox-2–only part of the image. (**C**) CAV1 (top) and SOX2 (bottom) staining in nontumor tissue control samples (healthy human hepatocytes). (**D**) The percentage of cells positive for caveolin-1 staining was calculated for each tumor section and segregated by grouping: complete response, progressive disease, or controls (healthy human hepatocytes). (**E**) The percentage of cells positive for Sox-2 staining was calculated for each tumor section and segregated by grouping: complete response, progressive disease, or controls. (**F**) The within-tumor section difference between percentages of caveolin-1–positive and Sox-2–positive cells for patients experiencing complete response or progressive disease in response to cetuximab treatment. Gray dots denote tumor sections that may be misclassified according to this metric. When available, multiple tumor sections are included from the same patient (total tumor sections = 16, total patients = 9). Original magnification, ×20 (**A**–**C**).

To investigate the clinical utility of our biomarker signature detected in HNSCC PDXs, we next assessed expression of signature proteins directly in tumors from cetuximab-treated patients with HNSCC with known clinical responses, as described previously (18). Immunohistochemistry was performed on 16 sections of a tumor microarray (TMA) generated from this cohort of 9 total cetuximab-treated patients, and protein expression was evaluated by determining the percentage of tumor cells positive for the marker of interest (Supplemental Figure 2 for images of tumor sections and Methods). Specifically, we assessed protein expression of caveolin-1 and Sox-2, the top 2 biomarkers from our signature. Given the wide spectrum of tumor responses to cetuximab in the clinical setting, we used clinical evaluation of complete response (CR) as a proxy for cetuximab sensitivity and progressive disease (PD) as representative of cetuximab resistance. In agreement with our PDX samples, we found that tumor tissue cores from patients with CR possessed higher caveolin-1 positivity and lower Sox-2 positivity than those from patients with PD (Figure 6, A–E, and Supplemental Table 6). Finally, we found a within-patient difference metric between caveolin-1 and Sox-2 — calculated as the difference between the percentage of positivity of caveolin-1 and Sox-2 in a patient’s tumor sample — to be a strong predictive metric of cetuximab response in these patients with HNSCC (Figure 6F).

## Discussion

This study presents a biomarker signature to predict cetuximab response with significant clinical implications for patients with HNSCC. Using an unbiased clustering approach allowed for separation of 2 distinct PDX clusters, which we hypothesized would demonstrate sensitivity or resistance to cetuximab based on differential expression of 2 sets of protein biomarkers. The predicted cetuximab responses were correctly validated in vivo for 88% (15 of 17) of HNSCC PDXs. Further interrogation of differential biomarker expression using a statistical classifier modeling approach revealed a robust 8-biomarker signature that was predictive of cetuximab response using either protein or mRNA expression inputs. The appearance of this signature at both the protein and mRNA levels indicates that it likely reflects a transcriptional program, possibly denoting a change in cell state or identity. The 8-gene signature was additionally validated using mRNA-sequencing data from an independent HNSCC PDX cohort. However, the drop in predictive power for caveolin-1 and Sox-2 at the mRNA level speaks to the possibility of some posttranslational stability or processing. Further refinement revealed caveolin-1 and Sox-2 protein measurements to retain high predictive accuracy for cetuximab response prediction, which we confirmed in patient samples using immunohistochemistry of a TMA from cetuximab-treated patients with HNSCC with known clinical responses.

The use of patient-specific molecular markers to guide precision medicine has been successful, with patients receiving genotype-directed agents demonstrating longer survival than those who did not receive targeted therapy (19–22). In colorectal cancer (CRC), cetuximab use is guided by the presence of RAS mutations (23), with KRAS alterations being a robust predictor of both adverse prognosis and cetuximab resistance (24, 25). Clinical guidelines advise the use of EGFR inhibitors only for CRC tumors lacking RAS mutations (26). However, evidence that KRAS alterations, which are rare in HNSCC (~1.4%), are a marker for cetuximab response in HNSCC is inconsistent, as are the data on the impact of EGFR alterations on cetuximab response, highlighting the paucity of molecular markers available to guide the use of cetuximab for HNSCC treatment (27, 28).

PDXs are a promising model for discovering novel biomarkers for therapy response, as they more accurately reflect the biological and clinical characteristics of primary human tumors than do cell lines (29, 30). One study of 25 CRC PDXs reported that a 147-gene RAS pathway transcriptomic signature significantly outperformed EGFR expression in predicting cetuximab response (31), supporting the development of multigene mRNA expression signatures as predictive biomarkers for therapy responses. Another study of 106 primary human CRC tumor samples and 59 PDXs aimed to identify a molecular signature predictive of cetuximab sensitivity in KRAS wild-type CRC (32). The researchers trained an SVM with RNA expression data and identified a 16-gene signature able to stratify cetuximab responders from nonresponders with high accuracy, outperforming the use of KRAS/NRAS/BRAF mutational status. Collectively, these studies support the use of PDXs as representative preclinical models in identifying biomarkers for precision cancer medicine. Of note, one limitation of the NOD/SCID γ mice used in this study is the lack of an intact immune system, rendering it impossible to assess the impact of the immune response in influencing tumor growth and drug response.

Recent trials of cetuximab in HPV-positive oropharyngeal squamous cell carcinoma (De-ESCALaTE and RTOG 1016) found that cetuximab combined with radiotherapy was associated with significantly worse overall survival and increased tumor recurrence compared with the combination of cisplatin and radiotherapy (16, 17). Interestingly, in our cohort, we observed that 14 of 15 HPV-positive PDXs were predicted to be cetuximab resistant (Figure 1B). However, while HPV status was clearly associated with predicted cetuximab response, it is notable that 22% (11 of 50) of the HPV-negative PDXs in the cohort were also predicted to be cetuximab resistant. In this study, we found that our biomarker signature significantly outperformed HPV status for cetuximab response prediction, as evidenced by Cramér’s V analysis (Figure 3D). This emphasizes the benefit of patient stratification based on molecular tumor characteristics rather than relying on binary HPV status.

Of all of the candidate biomarkers identified in our signature, Sox-2 and caveolin-1 demonstrated the largest overall change between the cetuximab-resistant and -sensitive HNSCC PDX clusters. Aberrant Sox-2 expression is associated with many malignancies and has well-characterized roles in tumor growth, metastasis, and drug resistance (33). In cutaneous squamous cell carcinomas, chromatin immunoprecipitation–sequencing experiments indicated that Sox-2 directly regulates gene networks promoting cancer stemness, proliferation, and cell survival (34). In HNSCC, PI3K signaling via mechanistic target of rapamycin (mTOR) increases SOX2 expression, resulting in the transcription of key cancer stem cell (CSC) genes, such as ALDH1A1 (11). HNSCC CSCs promote the resistance of HNSCC tumors to standard therapeutics, including cetuximab and docetaxel; in contrast, direct PI3K inhibitors substantially inhibit CSC-mediated tumorigenesis (11). Additionally, inhibition of PI3K signaling decreases Sox-2 protein levels in CSCs, while direct EGFR targeting does not, suggesting that PI3K/mTOR inhibition may successfully circumvent cetuximab resistance via downregulation of Sox-2 and modulation of downstream transcriptional programs (11). It would be interesting for future in vivo studies to assess Sox-2 levels in response to cetuximab treatment. Previous studies in lung cancer support Sox-2–mediated resistance to EGFR tyrosine kinase inhibitors. In vitro, treatment of EGFR mutant lung cancer cells with erlotinib induces Sox-2 expression, with SOX2 knockdown resulting in increased erlotinib-mediated apoptosis and delayed resistance to EGFR inhibition (35). In HNSCC, increased Sox-2 expression correlates with worse prognosis and disease recurrence (36). Notably, knockdown of SOX2 in HNSCC CSCs diminishes cellular self-renewal, invasive potential, and chemoresistance (14).

All together, our findings expand the knowledge base for designing future biomarker-guided clinical trials of cetuximab therapy, highlighting a potentially novel signature for cetuximab response based on molecular tumor profiling of either protein or mRNA. Remarkably, the 8-biomarker signature we present here demonstrates high accuracy for in vivo cetuximab response prediction using PDX samples, and a 2-protein signature consisting of caveolin-1 and Sox-2 retains high accuracy for predicting cetuximab response, both in PDXs and directly in samples from patients with HNSCC. Future studies should assess these findings in larger PDX and patient cohorts. In addition, further work is needed to determine whether these biomarkers mechanistically contribute to therapy response in HNSCC. However, even in the absence of detailed mechanistic understanding, the signature may successfully guide rational and personalized clinical decisions regarding cetuximab use for patients with HNSCC.

## Methods

### PDX generation.

PDXs were derived from patients with HNSCC upon written consent and were established as described previously (8). All PDXs were established in 5- to 6-week-old NOD.Cg-Prkdc^scid^ Il2rg^tm1Wjl^/SzJ mice (The Jackson Laboratory).

### Determining in vivo cetuximab response.

Ten predicted sensitive PDXs and seven predicted resistant PDXs were assessed for in vivo response to cetuximab. When the PDX tumor volume reached approximately 200 mm^3^, the mice were randomized into groups and treated with vehicle (saline) or 20 mg/kg cetuximab twice per week by intraperitoneal injection. Tumor dimensions were measured with calipers 2 times per week, and tumor volume was calculated using the formula length × width × width/2. Endpoint tumor volume for cetuximab-treated versus vehicle-treated PDXs was calculated on the final day of treatment for each PDX, which varied between day 15 and day 26. Cetuximab-sensitive PDXs were defined as those with at least a 50% reduction in normalized endpoint tumor volume in cetuximab-treated tumors relative to vehicle-treated tumors; the other PDXs were deemed cetuximab resistant.

### RPPA.

PDX samples were prepared and submitted as described previously (8), in accordance with procedures provided by the Functional Proteomics RPPA Core Facility of the University of Texas MD Anderson Cancer Center (Houston, Texas, USA). The RPPA Core Facility possesses a preselected set of 247 antibody probes quantifying total or modified proteins (i.e., phosphorylation) that have been demonstrated to be relevant to cancer disease progression. Median-centered log_2_ RPPA values for each protein of the samples was used in subsequent clustering and regression analyses.

### RNA-Seq.

Total RNA was extracted from 69 whole PDX tumors using the RNAeasy kit (Qiagen) following manufacturer’s instructions (only 65 were retained for downstream analyses). Total RNA was enriched for poly-A transcripts and sequenced using the NovaSeq 6000 platform (100PE) at the QB3-Berkeley Genomics center at the University of California Berkeley. Following sequencing, the unaligned reads contained a mix of human stroma, mouse germline, viral, and ostensibly other sequences. Hence, prior to mapping to human reference and expression counting, reads were classified to a probable source. For convenience, each sample had been previously split between 2 fastq files. Adapters were trimmed and reads failing QC were removed using fastp (v0.20.0). Reads were classified as “human,” “mouse,” “both,” “neither,” and “ambiguous” using Xenome (v1.0.0), a k-mer index–based pseudoaligner, with each sample’s split files classified independently of each other. Only reads classified as uniquely human progressed to the human expression pipeline. Uniquely human reads were merged into a single fastq file for each sample and next aligned against a ribosomal reference using bwa, removing rRNA matches. Finally, remaining reads were mapped against an Ensembl GRCh38.90 reference. Expression counts were estimated using RSEM (v1.3.1). mRNA-sequencing data (in units of FPKM) was median centered across patients by dividing by the median expression value for each gene. The log_2_ of these values was then calculated to generate the final RNA expression values used for subsequent analyses. Raw and processed mRNA-sequencing data can be accessed at GEO under the accession GSE183881.

### Hierarchical unbiased clustering analysis.

We used an unbiased and unsupervised hierarchical clustering analysis to cluster the 65 PDX tumors using 247 median-centered log_2_ intensities from RPPA antibody probes. The “ward” algorithm in MATLAB (R2018a) was used for this analysis. Once completed, visual inspection revealed 2 predominant biomarker groups with clear and strong differential expression between the 2 clusters.

### Enrichment analysis and network creation.

Corresponding genes were extracted from biomarker groups 1 and 2. MSigDB was used to perform gene set enrichment analysis against Reactome (37), KEGG (38), Biocarta, and PID pathway terms to probe broad functionality of collective lists of genes potentially responsible for cetuximab resistance (c2.cp.v7.1.symbols.gmt). The top 10 most-enriched terms with FDR < 0.05 were kept (Figure 1). To create interaction networks, subgraphs were created using genes extracted from biomarker groups 1 and 2. Functional interactions were extracted from the ReactomeFI network. Physical interactions were extracted from a union of several protein-protein interaction networks, including BioPlex, IRefIndex, Mentha, Human Interactome, and Human Protein Reference Database (HPRD), as previously described (39). The union of functional and physical interactions was depicted for biomarker group 1 and 2 separately (Figure 1).

### Defining and validating biomarker sets using SVM classifier.

For each RPPA probe, the average of the log_2_ median-centered values for each PDX cluster (CTX-R and CTX-S) was calculated. Next, the fold changes between the 2 patient clusters were calculated by taking the difference between their means (CTX-R – CTX-S). The statistical significance of the difference was calculated using Student’s *t* test. Only RPPA probes with a *P* value of < 0.01 were considered for subsequent analyses. Absolute value fold change thresholds were imposed in 0.1 increments from 0.1 to 1.5, and RPPA probe sets were extracted at each threshold increment, resulting in 15 total biomarker sets. Each biomarker set was then used to isolate biomarkers for training a SVM in MATLAB, excluding the 17 PDXs that had undergone experimental testing for in vivo cetuximab response. These 17 were then used to test the performance of the trained model. The classifier’s performance was evaluated by plotting receiver operator characteristics and precision-recall curves and extracting the area under these curves. To test the mRNA-sequencing data, the same approach was used as above. SVM models were always trained on the RPPA data and subsequently tested using expression values from the median-centered log_2_ RNA-Seq data. The same approach was used to evaluate the performance of the RNA-Seq data from Keysar et al. (9). The training data used was also the RPPA protein data from this study. This trained model was tested using the RNA-Seq data from Keysar et al. from PDXs with known cetuximab response.

### Immunohistochemistry and imaging analysis.

TMA slides were stained with the Sox2 (D1C7J) and caveolin-1 (D46G3) from CST and cytokeratin 5,6 (RM341) from Invitrogen. Sox2 and caveolin-1 were detected by purple and cytokeratin 5,6 by yellow chromogen. Nucleic acid was stained using DAPI (blue). All antibodies were optimized and stained on a Ventana Discovery Ultra autostainer using Discovery reagents (Ventana Medical Systems). Slides were then scanned in bright-field mode with Plan-Apochromat 20×/0.8 M27 objective and HV-F202SCL CCD camera (Hitachi). Scanned images were imported to QuPath (40) for cell quantification. First, cell nuclei were detected with the StarDist algorithm (41). Then QuPath’s default algorithm was used to determine the cell boundaries. Mean intensity of each cell was calculated. Based on an intensity threshold, cells were labeled as either positive or negative for each marker. The threshold value was determined by comparing the staining pattern with that of the optimized antibody on control tissue (healthy human liver sections). Automated quantification of CAV1 and SOX2 positivity was done using all cells in each section, not simply those labeled positive by cytokeratin 5,6 staining. Segmentation using cytokeratin 5,6–positive staining is a difficult manual process prone to human error. However, we did perform manual segmentation for 1 set of images (CAV1-positive images) in order to compare if the results were significantly different than an automated analysis of the entire section. We found the 2 approaches to be highly correlated (*R* = 0.98) in terms of the percentage of CAV1-positive cells per section. Due to this, we opted to proceed with the automated whole-section analysis pipeline as described above.

### Statistics.

To calculate differential expression of RPPA probe intensities, a 2-tailed Student’s *t* test was used, and a *P* value cutoff of 0.05 was applied. For the enrichment analysis, the top 10 most-enriched terms were determined using a 1-tailed Fisher’s exact *t* test, and a cutoff of FDR < 0.05 was applied.

### Study approval.

All experiments using mice were approved by the Institutional Animal Care and Use Committee of the University of California, San Francisco (protocol no. AN173372). The use of the human patient tumor sections was approved through Tissue Bank IRB no. 14-15342.

## Author contributions

This study was conceived by JRG, DEJ, MB, RHL, NEB, and RAO. The study was supervised by JRG, DEJ, DLS, NJK, and NEB. Computational analyses were performed by MB and RHL. The manuscript was written by RHL, MB, JRG, DEJ, NEB, and RAO. In vivo animal experiments were performed and/or analyzed by HL, RHL, NEB, and RAO. RPPA data were generated by UD and GBM. PDXs were characterized by NEB, KN, TRZ, SBK, and AJ. RNA-Seq analysis was performed by ABO, RR, and AH. Immunocytochemistry was performed and/or analyzed by JB, MN, and SV.

## Supplementary Material

Supplemental data

Supplemental table 1

Supplemental table 2

Supplemental table 3

Supplemental table 4

Supplemental table 5

Supplemental table 6

## Version 1 09/21/2021

In-Press Preview

## Version 2 10/22/2021

Electronic publication
