# Supplementary material for: Caveolin-1 and Sox-2 are predictive biomarkers of cetuximab response in head and neck cancer
Source: JCI Insight. 2021 Oct 22;6(20):e151982. doi: 10.1172/jci.insight.151982 (PMC8564908; doi:10.1172/jci.insight.151982)
Supplement: Supplemental data [file jciinsight-6-151982-s250.pdf]

SUPPLEMENTARY INFO

Supplemental Table 1. Demographic and clinical characteristics of the 65 HNSCC patients from whom this PDX collection was derived. Includes site of primary tumor, American Joint Committee on Cancer (AJCC) staging, age, sex, and HPV status.

Supplemental Table 2. Full PDX characteristics table.

Supplemental Table 3. Median centered RPPA data from PDX samples, biomarker groups, and statistical comparison between PDX clusters.

Supplemental Table 4. Temporal PDX tumor volume measurements.

Supplemental Table 5. Median centered RNA sequencing data from PDX samples.

Supplemental Table 6. Quantification from patient tumor sample histology.

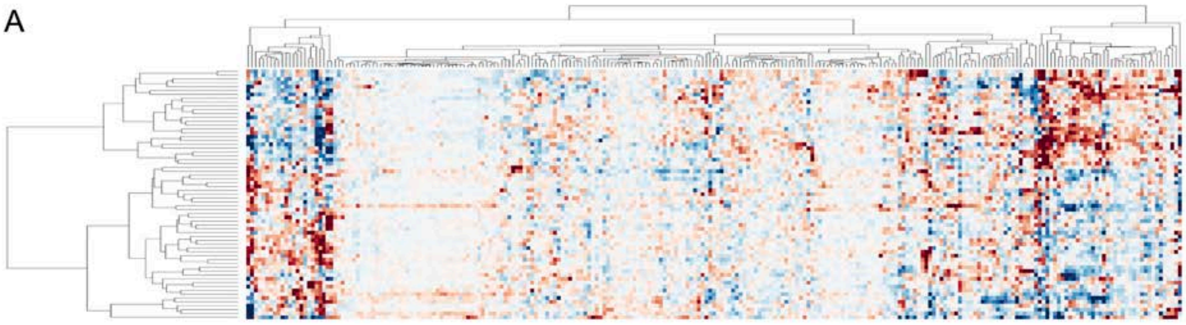

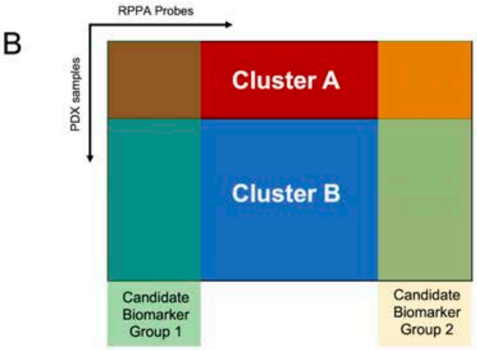

C

Group 1	Group 2	
Connexin43RC	IGFBP2RV	Rad50RV
NDRG1_pT346RV	Sox2RV	BimRV
LDHARC	PARRC	XRCC1RC
x1433zetaRV	Claudin7RV	RBM15RV
Jagged1RV	Rab25RV	Wee1_pS642RC
PCadherinRC	ECadherinRV	cdc2_pY15RC
VEGFR2RV	bCateninRV	FOXM1RV
IRS1RV	PLK1RC	GCN5L2RV
PaxillinRC	CDK1_pT14RC	TFAMRV
MMP14_EP1264Y_RN	CyclinB1RV	CoxIVRV
PKCaRV	cdc25CRV	HistoneH3RV
GlutaminaseRC	PARPRV	DMHistoneH3RV
HeregulinRV	MSH6RC	SLC1A5RC
Cox2RC	Wee1RC	TFRCRV
DUSP4RV	ARID1ARC	HSP70RC
PDGFRbRV	x53BP1RV	Caspase7cleavedRC
YAPRC	BRD4RV	ZAP70RC
YAP_pS127RV	Pdcd4RC	CD4RV
Caveolin1RV	ATMRV	STINGRV
AxlRV		
FibronectinRV		
S6_pS240_S244RV		
S6_pS235_S236RV		

Figure S1. Full hierarchical clustering results and biomarker group definitions.
(A) Complete clustergram for unbiased hierarchical clustering using the "ward" algorithm (evaluated in MATLAB) for all 247 probes measured across the 65 PDXs. Rows indicate PDX samples and columns indicate RPPA probes. (B) Schematic representation of PDX division into two cluster subtypes, defined by differential expression of Biomarker Groups 1 and 2. (C) Full list of proteins belonging to Biomarker Group 1 and Group 2.

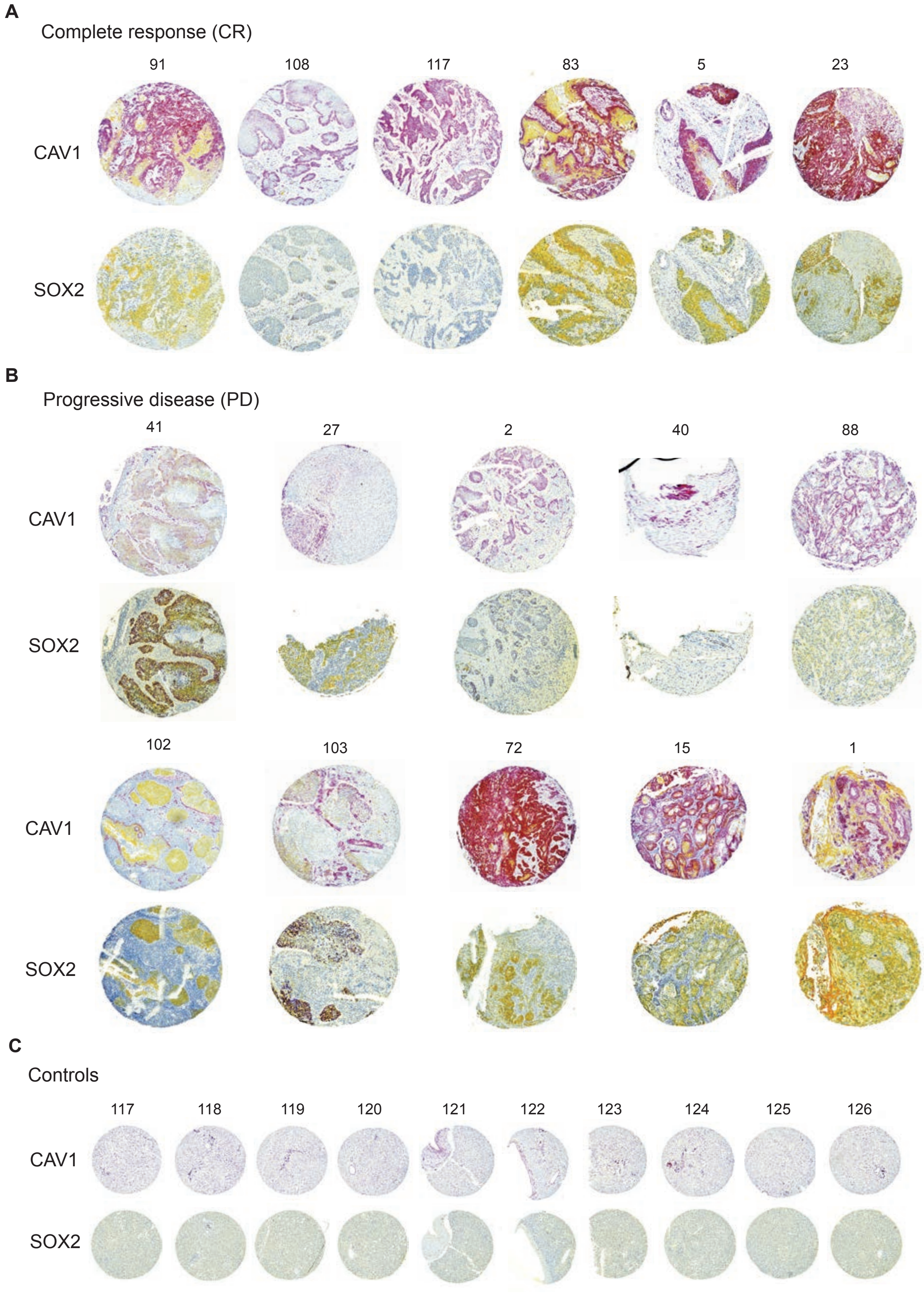
A
Complete response (CR)
91
108
117
83
5
23
CAV1
SOX2
B
Progressive disease (PD)
41
27
2
40
88
CAV1
SOX2
102
103
72
15
1
CAV1
SOX2
C
Controls
117
118
119
120
121
122
123
124
125
126
CAV1
SOX2

Figure S2. Images of stained patient tumor samples.

(A) Tumor sections from patients who experienced a complete response (CR) to cetuximab treatment. CAV1 (purple) is stained on the top and SOX2 (purple) is stained on the bottom. Cytokeratin 5,6 is in yellow on both. (B) Tumor sections from patients who experienced progressive disease (PD) (i.e. resistance) to cetuximab treatment. CAV1 (purple) is stained on the top and SOX2 (purple) is stained on the bottom. Cytokeratin 5,6 is in yellow and nucleic acid (nuclei) is stained using DAPI (blue) on both. (C) CAV1 (top) and SOX2 (bottom) stained non-tumor hepatocyte controls. Numbering corresponds to core number (see Table S6 for relationship between core number and patient number).
