# Supplementary material for: Caveolin-1 and Sox-2 are predictive biomarkers of cetuximab response in head and neck cancer
Source: JCI Insight. 2021 Oct 22;6(20):e151982. doi: 10.1172/jci.insight.151982 (PMC8564908; doi:10.1172/jci.insight.151982)
Supplement: Supplemental table 1 [file jciinsight-6-151982-s251.pdf]

| | | Total | Predicted Sensitive | Predicted Resistant | % Predicted Resistant |
|---|---|---|---|---|---|
| Primary Site | Oral Cavity | 35 | 32 | 3 | 9% |
| | Pharynx | 21 | 5 | 16 | 76% |
| | Larynx | 9 | 3 | 6 | 67% |
| AJCC Stage | I/II | 14 | 9 | 5 | 36% |
| | III | 14 | 9 | 5 | 36% |
| | IV | 37 | 22 | 15 | 41% |
| Age | ≤55 | 24 | 16 | 8 | 33% |
| | 56-60 | 17 | 11 | 6 | 35% |
| | 61-67 | 10 | 5 | 5 | 50% |
| | ≥68 | 14 | 8 | 6 | 43% |
| Sex | Male | 53 | 31 | 22 | 42% |
| | Female | 12 | 9 | 3 | 25% |
| HPV Status | Positive | 15 | 1 | 14 | 93% |
| | Negative | 50 | 39 | 11 | 22% |

**Supplemental Table 1.** Demographic and clinical characteristics of the 65 HNSCC patients from whom this PDX collection was derived. Includes site of primary tumor, American Joint Committee on Cancer (AJCC) staging, age, sex, and HPV status.
